# Supplementary material for: Cellular and genetic drivers of RNA editing variation in the human brain
Source: Nat Commun. 2022 May 30;13:2997. doi: 10.1038/s41467-022-30531-0 (PMC9151768; doi:10.1038/s41467-022-30531-0)
Supplement: Supplementary file 1 — Supplementary Information [file 41467_2022_30531_MOESM1_ESM.pdf]

Supplementary Information for:

Cellular and genetic drivers of RNA editing variation in the human brain

Winston H. Cuddleston[1,2,3,4*], Junhao Li[5*], Xuanjia Fan[1,2,3,4], Alexey Kozenkov[1,6], Matthew Lalli[1,3], Shahrukh Khalique[1,6], Stella Dracheva[1,6], Eran A. Mukamel[5], Michael S. Breen[1,2,3,4]

[1]Department of Psychiatry at Mount Sinai, New York, New York, 10029, USA
[2]Department of Genetics and Genomic Sciences at Mount Sinai, New York, New York, 10029, USA.
[3]Seaver Autism Center for Research and Treatment at Mount Sinai, New York, New York, 10029, USA.
[4]Pamela Sklar Division of Psychiatric Genomics at Mount Sinai, New York, New York, 10029, USA.
[5]Department of Cognitive Science, University of California, San Diego, La Jolla, CA 92037, USA.
[6]James J Peters VA Medical Center, Bronx, New York, 10468, USA.

Co-first authorship*
Correspondence to: michael.breen@mssm.edu

Supplementary Figures 1-19:

Supplementary Figure 1. RNA hyper-editing across cell types.
Supplementary Figure 2. Annotation of A-to-G sites detected in only one cell type.
Supplementary Figure 3. RNA editing sites are commonly detected on low-to-moderately expressed genes.
Supplementary Figure 4. Quantifying variance in RNA editing rates by known factors.
Supplementary Figure 5. RNA editing variation and RNA binding protein analysis.
Supplementary Figure 6. Synaptic gene-set enrichment analysis.
Supplementary Figure 7. Overlap of differentially edited sites and differentially expressed genes.
Supplementary Figure 8. PhastCons scores by genic regions.
Supplementary Figure 9. Validation of cell-specific recoding events in independent samples
Supplementary Figure 10. RNA editing sites as a function of gene length.
Supplementary Figure 11. tSNE of snRNA-seq.
Supplementary Figure 12. Read bias distribution of FANS and scRNA-seq.
Supplementary Figure 13. Correlation of AEI with known factors in GTEx data.
Supplementary Figure 14. Principal component analysis on features of RNA editing in the brain
Supplementary Figure 15. Variance in RNA editing site detection per donor in bulk GTEx tissues.
Supplementary Figure 16. Annotation of sites detected in bulk GTEx brain tissue.
Supplementary Figure 17. Absolute effect sizes of edQTLs across GTEx brain regions
Supplementary Figure 18. edQTL discovery as a function of sample size
Supplementary Figure 19. eSite discovery in Park et al., 2021.

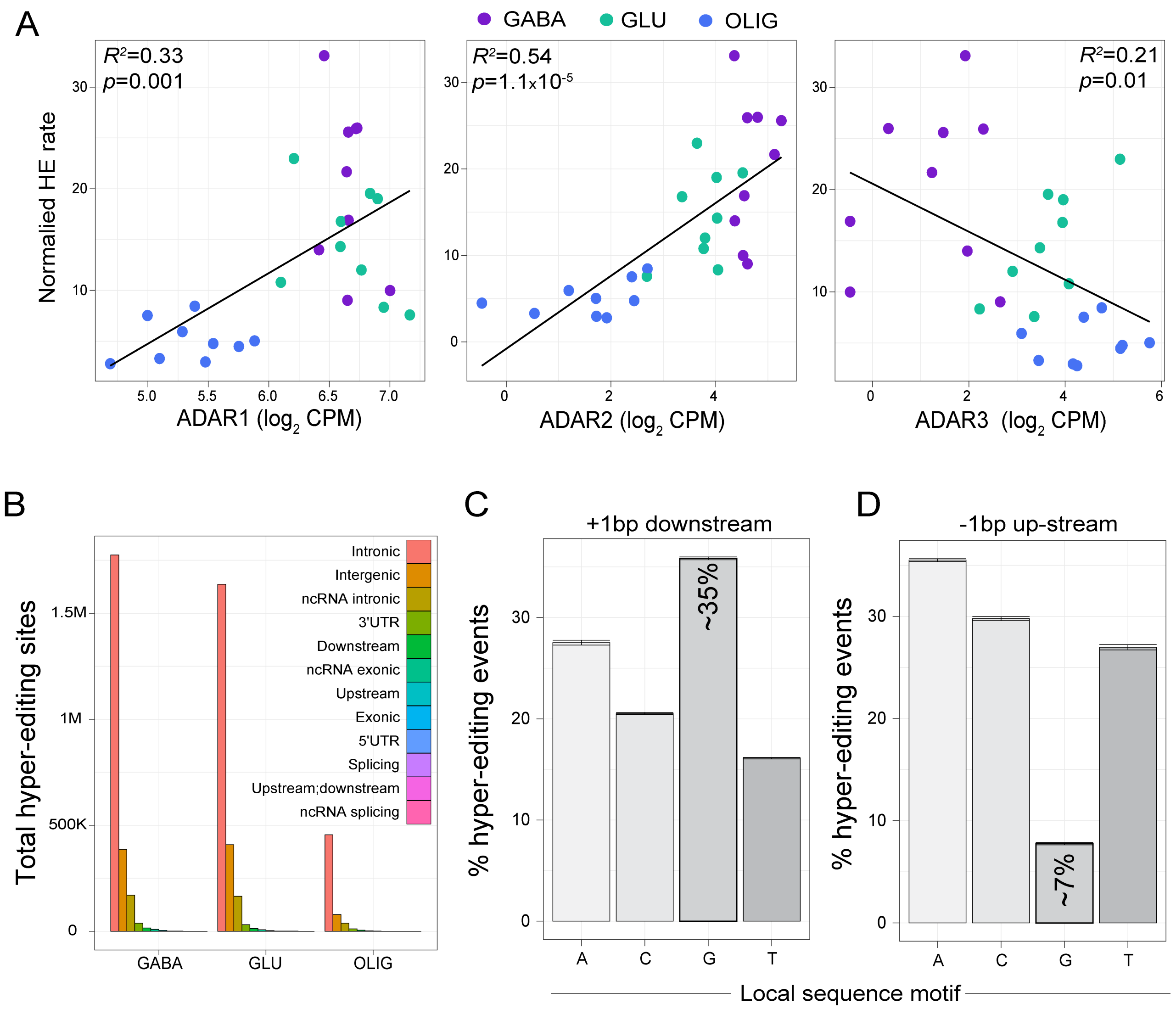

Supplementary Figure 1. RNA hyper-editing across cell types. **(A)** The amount of normalized hyper-editing sites per million mapped bases (normalized hyper-editing (HE) rate, x-axis) variance explained by expression of *ADAR1* (left), *ADAR2* (center), and *ADAR3* (right) was evaluated by Pearson's correlation coefficient. *R*-squared values are reported and no adjustments were made for multiple comparisons. (**B**) The total number of hyper-editing sites (y-axis) by genic region per cell type (x-axis). Hyper-editing sites were scrutinized for conserved local sequence motifs, that is (**C**) enrichment of guanosine +1 bp downstream **(D)** and depletion of guanosine -1 bp upstream of the target adenosine. Values are aggregated across all three cell types. FANS purified nuclei from the same prefrontal cortex samples were used in all panels (*n*=9 biologically independent samples).

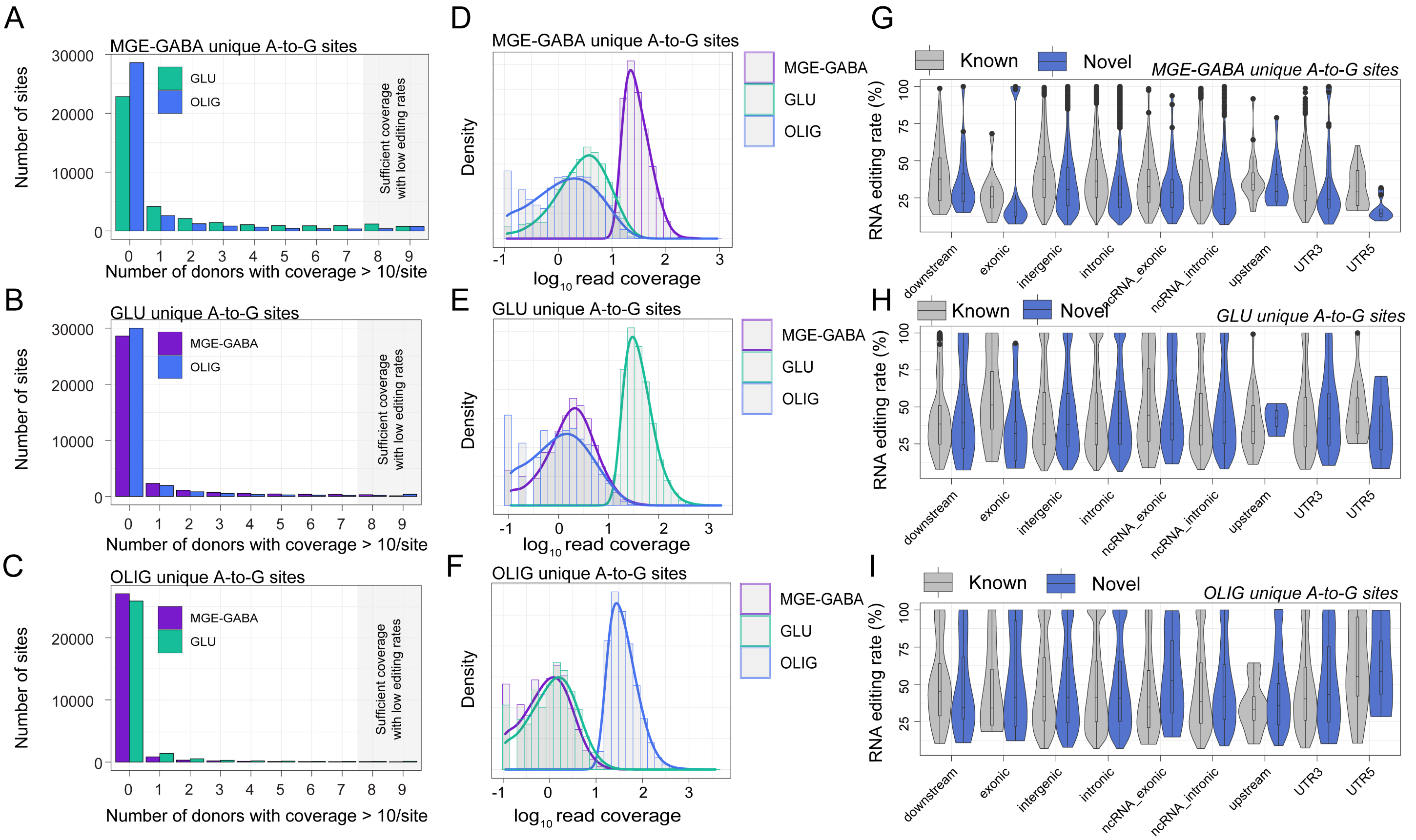

Supplementary Figure 2. Annotation of A-to-G sites detected in only one cell type. The number of donors (out of 9 per cell type) with a coverage of at least 10 reads for sites uniquely classified as cell type-specific in **(A)** MGE-GABA, **(B)** GLU, and **(C)** OLIG cells. Read coverage plots for sites uniquely classified as cell type-specific in **(D)** MGE-GABA, **(E)** GLU, and **(F)** OLIG cells. Plots demonstrate that sites uniquely detected per each cell type are largely associated with differences in cell type-associated read coverage differences. RNA editing levels for both known and novel A-to-G cell-specific sites parsed by genic region for **(G)** MGE-GABA, **(H)** GLU, and **(I)** OLIG cells. FANS purified nuclei from the same prefrontal cortex samples were used in all panels (*n*=9 biologically independent samples). Box plots show the medians (horizontal lines), upper and lower quartiles (inner box edges), and 1.5 × the interquartile range (whiskers).

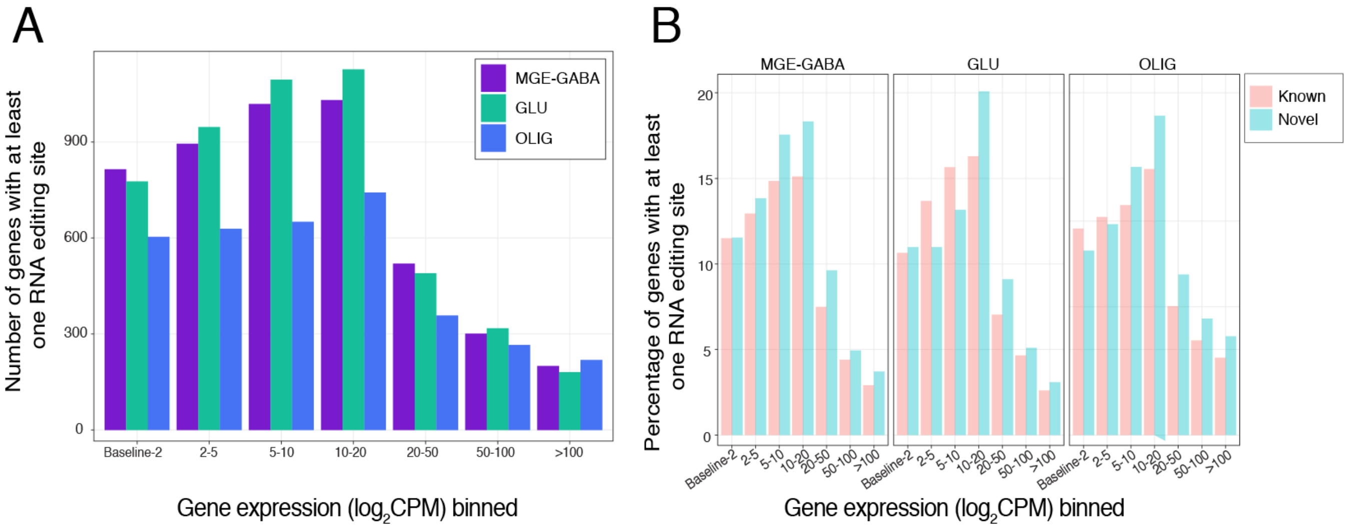

Supplementary Figure 3. RNA editing sites are commonly detected on low-to-moderately expressed genes. (A) The total number of genes with at least one RNA editing site (y-axis) relative to the corresponding expression level ($\log_2$CPM) of the gene (x-axis). **(B)** The percentage of genes with at least one RNA editing site (y-axis) according to binned gene expression (x-axis) parsed by novel sites and known sites detected in the REDiportal database. FANS purified nuclei from the same prefrontal cortex samples were used in all panels (*n*=9 biologically independent samples).

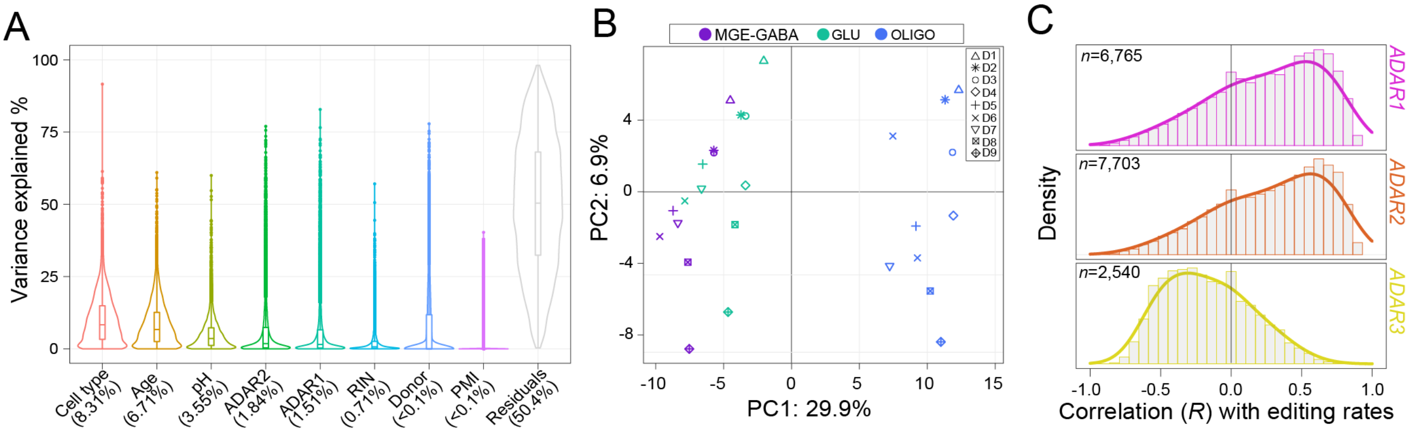

Supplementary Figure 4. Quantifying variance in RNA editing rates by known factors. Sites commonly detected across all cell types were leveraged for the analyses presented in all current panels (*n*=15,221 sites). **(A)** Mixed linear models were used to compute variance explained (%, y-axis) by several known factors and FANS-derived cell population (cell type) explained the largest source of variance (8.31% median). Box plots show the medians (horizontal lines), upper and lower quartiles (inner box edges), and 1.5 × the interquartile range (whiskers). **(B)** Principal component analysis of all donors (*n* = 9) stratifies neuronal (MGE-GABA, GLU) from non-neuronal (OLIG) cells. Samples are shaped differently based on donor (D) as a repeated measure, variance explained by PC2. **(C)** Density plot distributions illustrate the number of A-to-G editing sites which correlate with ADAR expression across all cell-types according to Pearson's correlation coefficient (x-axis). Associations between *ADAR* expression and editing levels (*n*=15,221 sites) were computed using a moderated t-test and adjusted for multiple comparisons using the Benjamini-Hochberg method. The total number of sites that are significantly associated (FDR P-value < 0.05) with ADAR expression are displayed in the top left corner for the gene encoding each enzyme. All data are presented in Supplementary Table 3.

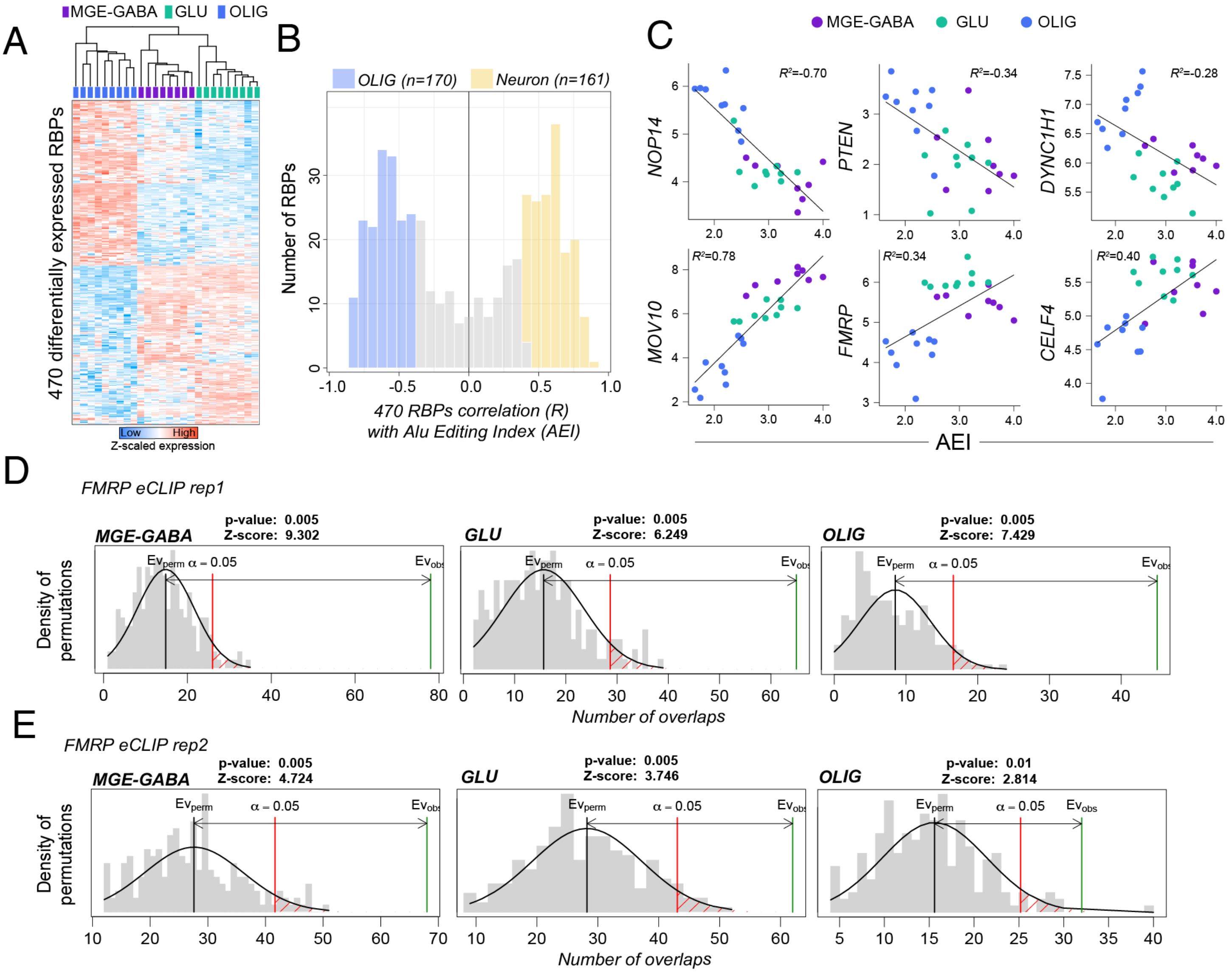

Supplementary Figure 5. RNA editing variation and RNA binding protein analysis. (A) Heatmap displaying the relative expression of 470 differentially expressed RNA binding proteins (RBPs) between MGE-GABA (purple) and GLU (green) neurons relative to OLIG (blue) cells. **(B)** Correlation of 470 RBPs with the AEI measurement across all cell types depicting a 161 RBPs strongly associated with the AEI in neuronal cells (MGE-GABA and GLU) and 170 RBPs strongly associated with the AEI in OLIG cells. Analyses were adjusted for repeated measures using Benjamini-Hochberg method. (**C**) Example of three RBPs strongly associated with the AEI in OLIG cells (top) and three RBPs strongly associated with the AEI in neuronal cells (bottom). Genomic coordinates for all cell type-associated RNA editing sites were assessed for enrichment for FMRP eCLIP binding sites across two technical replicates (**D-E**). The regioneR R package was used test overlaps of genomic regions based on permutation sampling. We repeatedly sampled random regions from the genome1000 times, matching size and chromosomal distribution of the region set under study. By re-computing the overlap with FMRP binding sites in each permutation, statistical significance of the observed overlap was computed.

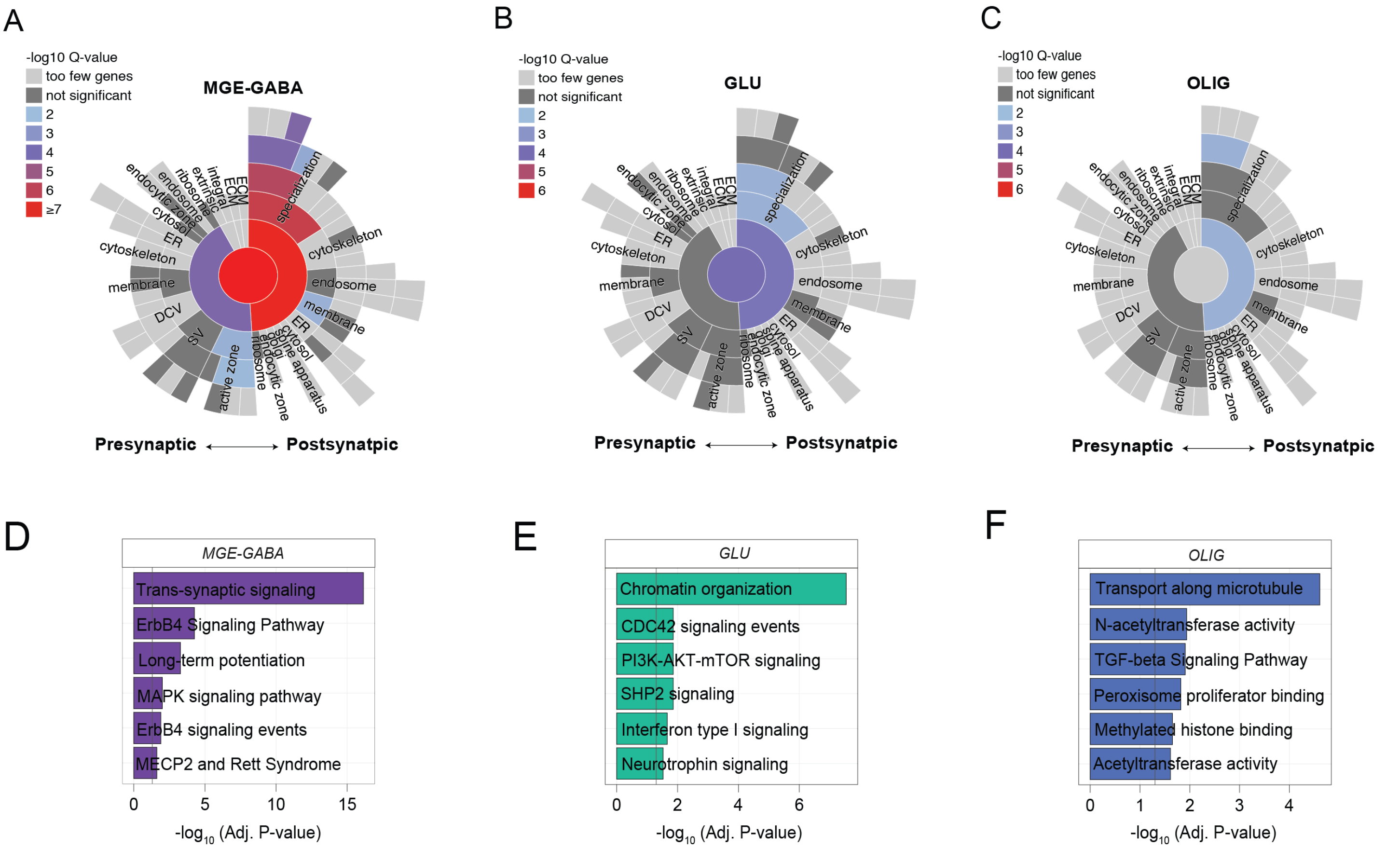

Supplementary Figure 6. Synaptic gene-set enrichment analysis. SynGO sunburst plots portray synaptic gene ontology enrichment associated with cell-type enriched RNA editing sites in **(A)** MGE-GABA, **(B)** GLU, and **(C)** OLIG cells. The top six cell-specific pathways (i.e. uniquely enriched for one cell type only) for the genes encompassing identified editing sites for in **(D)** MGE-GABA, **(E)** GLU, and **(F)** OLIG cells.

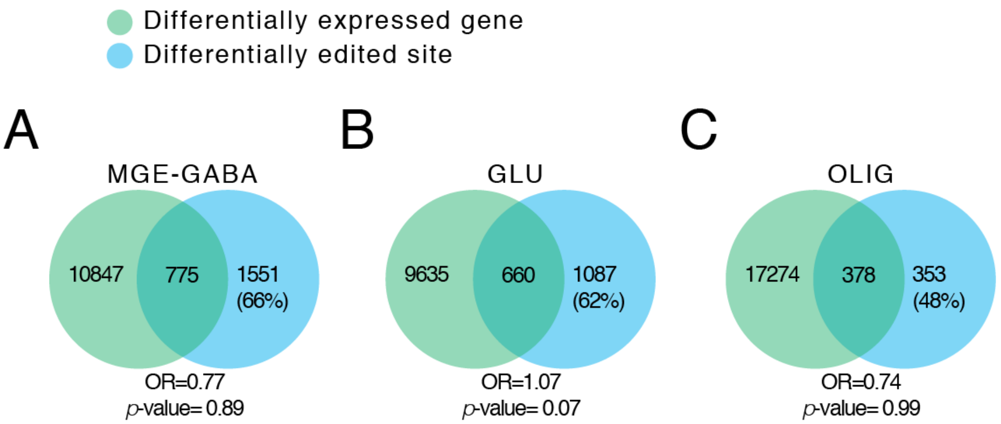

Supplementary Figure 7. Overlap of differentially edited sites and differentially expressed genes. Cell-specific gene expression was defined as genes with significantly higher expression (FDR P-value < 0.05) in one cell type relative to other cell types. A one-sided Fisher's Exact Test and an estimated odds-ratio (OR) was used to compute the significance of the overlap between cell-specific gene expression and genes harboring editing sites with cell-specific editing rates. We observed (**A**) ~66% of differentially edited sites in MGE-GABA nuclei were not explained by differential gene expression, (**B**) ~62% of differentially edited sites in GLU nuclei were not explained by differential gene expression, and (**C**) ~48% of differentially edited sites in OLIG nuclei were not explained by differential gene expression.

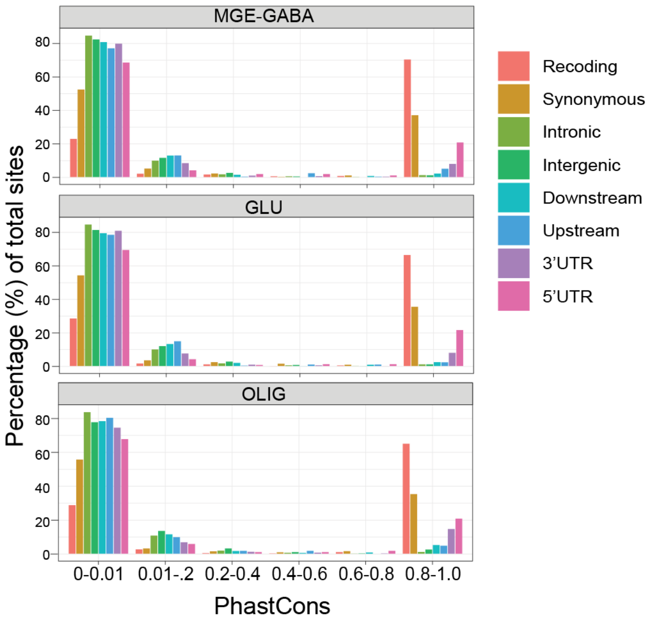

Supplementary Figure 8. PhastCons scores by genic regions. Editing sites reported for all three cell types binned by genic region and PhastCons, highlighting an increased proportion of highly conserved recoding sites across all three cell types, with less conservation in editing sites throughout other genic regions. FANS purified nuclei from the same prefrontal cortex samples (*n*=9 biologically independent samples).

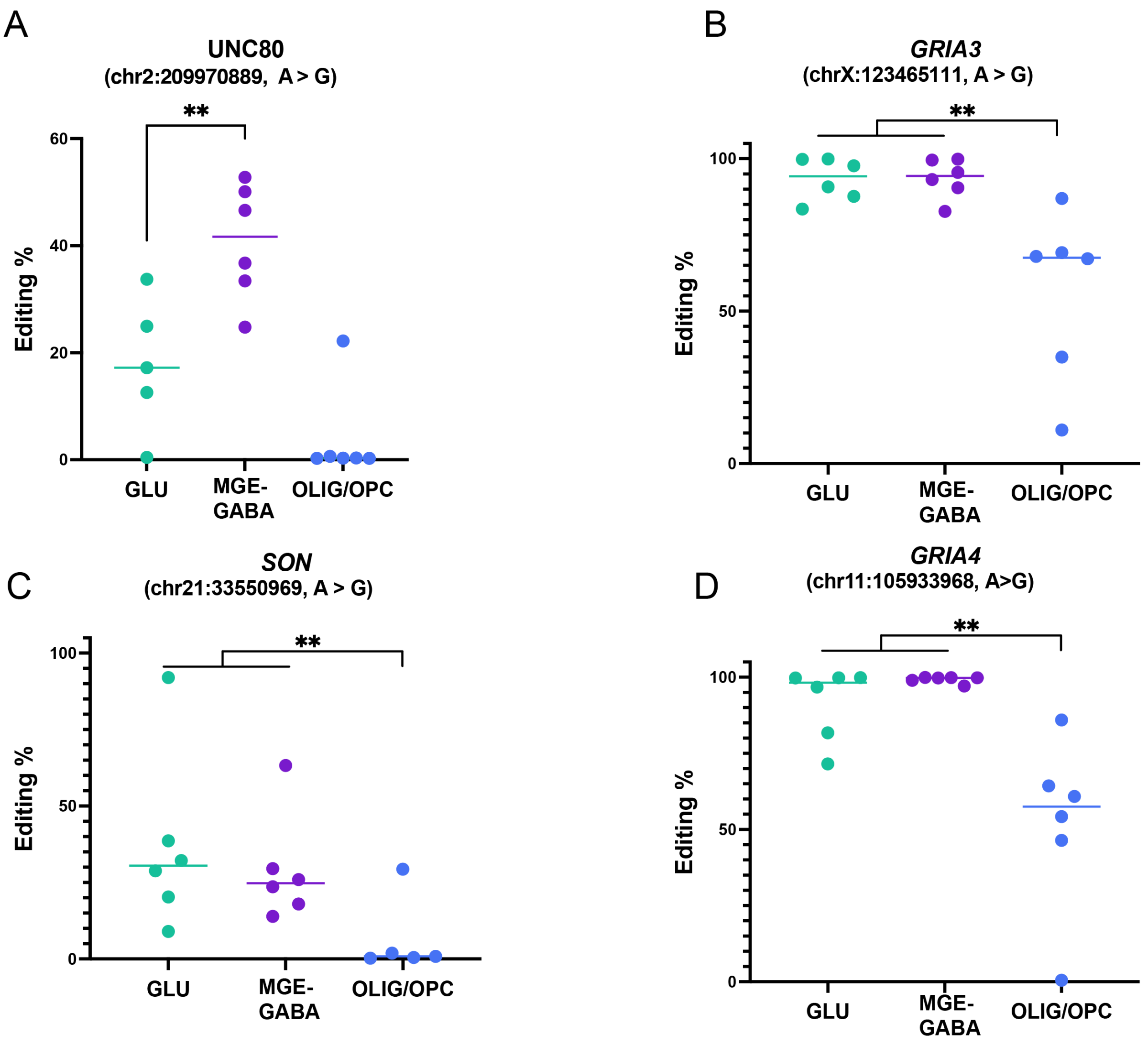

Supplementary Figure 9. Validation of cell-specific recoding events in independent samples. Four recoding sites were validated in independent OFC samples from healthy age matched donors (*n*=6 biologically independent samples) (*see Supplementary Table 6*). Editing levels were tested for four RNA recoding sites in (**A**) *UNC80*, (**B**) *GRIA3*, (**C**) *SON*, and (**D**) *GRIA4*, and editing levels highest in neurons relative to oligodendrocytes. Data are presented as median values (horizontal lines). rhAmpSeq targeted amplicon sequencing kit was used in two rounds of PCR amplification to obtain the targeted PCR products (PCR1) and to introduce unique indexes for multiplex sequencing (PCR2), according rhAMPSeq protocol (*see Methods*). Two-sided regression analyses were used to compute significance. No adjustments were made for multiple comparisons. Double asterisks reflect *p*-values less than 0.05. Exact p-values are provided in Supplementary Table 6.

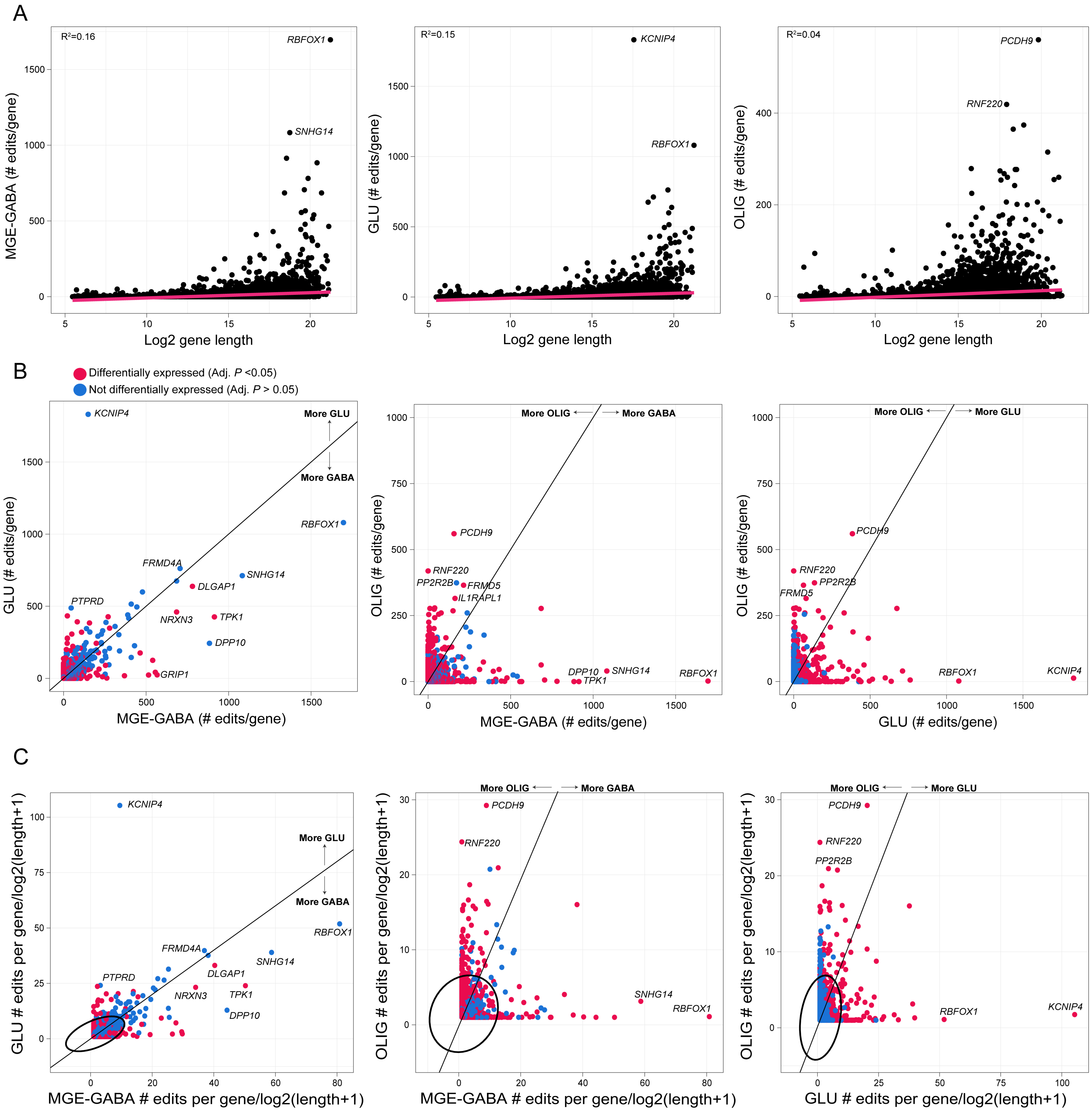

Supplementary Figure 10. RNA editing sites as a function of gene length. (A) Gene length as a function of the total number of selective RNA editing sites across all MGE-GABA (left), GLU (center) and OLIG cells (right). **(B)** Pairwise comparisons of concordance for the number of RNA editing sites per gene between two cell types – MGE-GABA vs. GLU (left), MGE-GABA vs OLIG (center), GLU vs. OLIG (right). **(C)** Pairwise comparisons of concordance for the number of RNA editing sites by gene divided by log2 of gene length between two cell types – MGE-GABA vs. GLU (left), MGE-GABA vs OLIG (center), GLU vs. OLIG (right). Outlier genes were classified as genes enriched with RNA editing sites and are denoted as those outside the 99% confidence intervals from the grand mean. In each panel, a linear regression is plotted and no adjustments were made for multiple comparisons. FANS purified nuclei from the same prefrontal cortex samples were used in all panels (n=9 biologically independent samples).

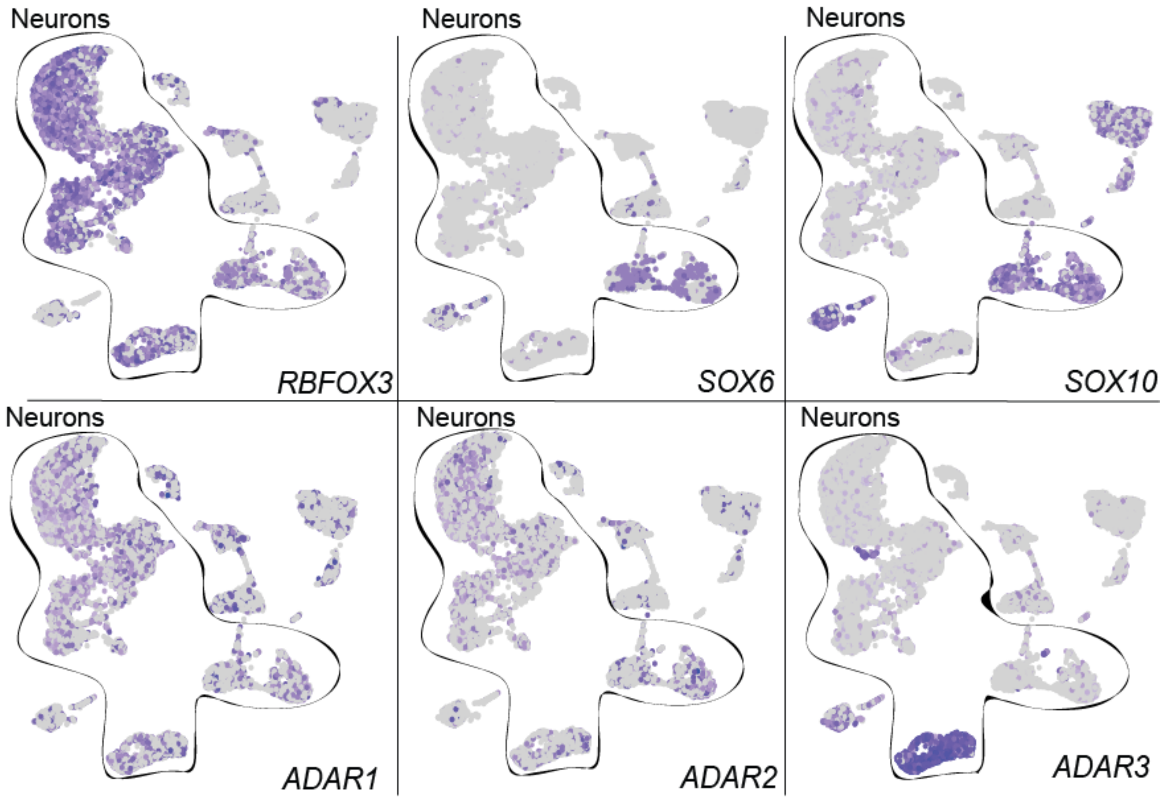

Supplementary Figure 11. tSNE of snRNA-seq. Cells were examined for expression markers: GLU (RBFOX3+/SOX6-), MGE-GABA (RBFOX3+/SOX6+), and OLIG (RBFOX3-/SOX10+) (top row) and *ADAR1*, *ADAR2* and *ADAR3* expression (bottom row). Both excitatory and inhibitory neuronal populations were identified and manually outlined in black, and all remaining glial and endothelial cell populations lay outside the black outline.

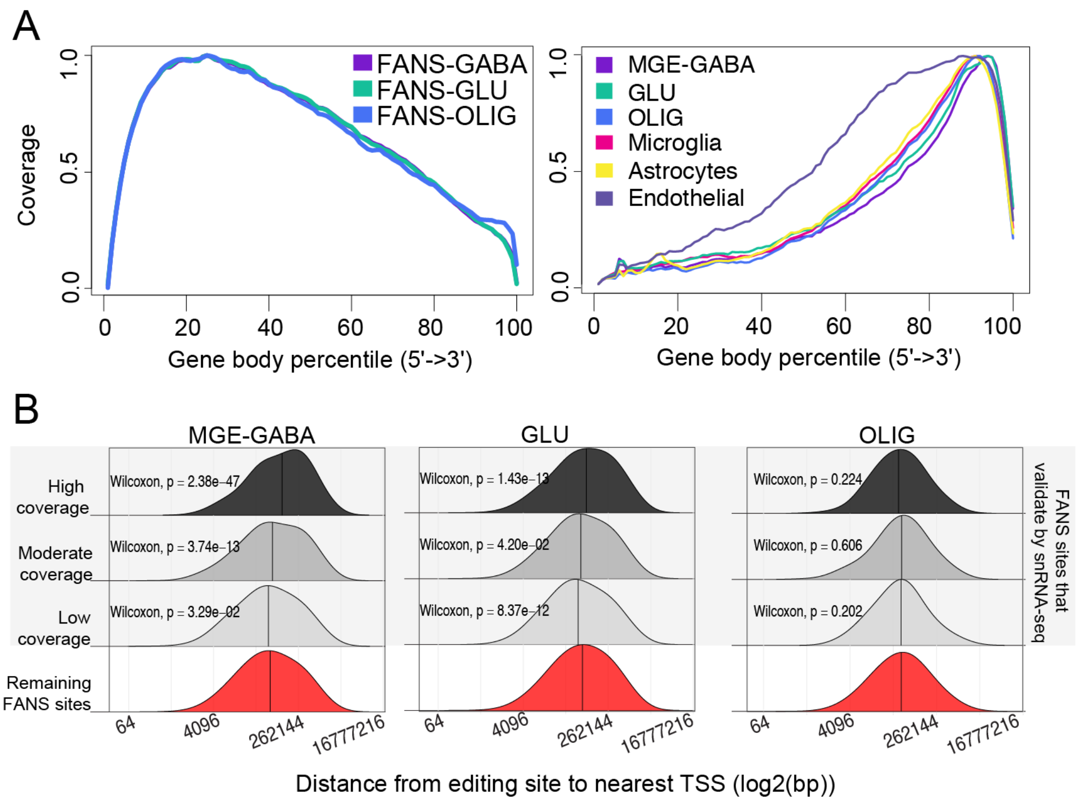

Supplementary Figure 12. Read bias distribution of FANS and scRNA-seq. (**A**) RseqQC computed RNA-seq read coverage over the entire gene body for all transcripts for the FANS RNA-seq data (right) and the snRNA-seq pools (left). The average coverage rates across all replicates per cell type are plotted. (**B**) Sites that validate by snRNA-seq were evaluated for 3' bias and were binned into three groups based on total snRNA-seq read coverage: 1) high coverage (the first quintile of coverage); 2) moderate coverage (the second, third and fourth quintile of coverage); and 3) low coverage (the fifth quintile of coverage) (y-axes). The distance between each site and the transcription start site (TSS) was measured (x-axis) for MGE-GABA (left), GLU (center) and OLIG (right). Two-sided Mann Whitney-U tests were used to examine significant differences in distances from the TSS for all binned sites that validate by snRNA-seq relative to sites without validation, whereby sites with higher read coverage show a greater 3' bias.

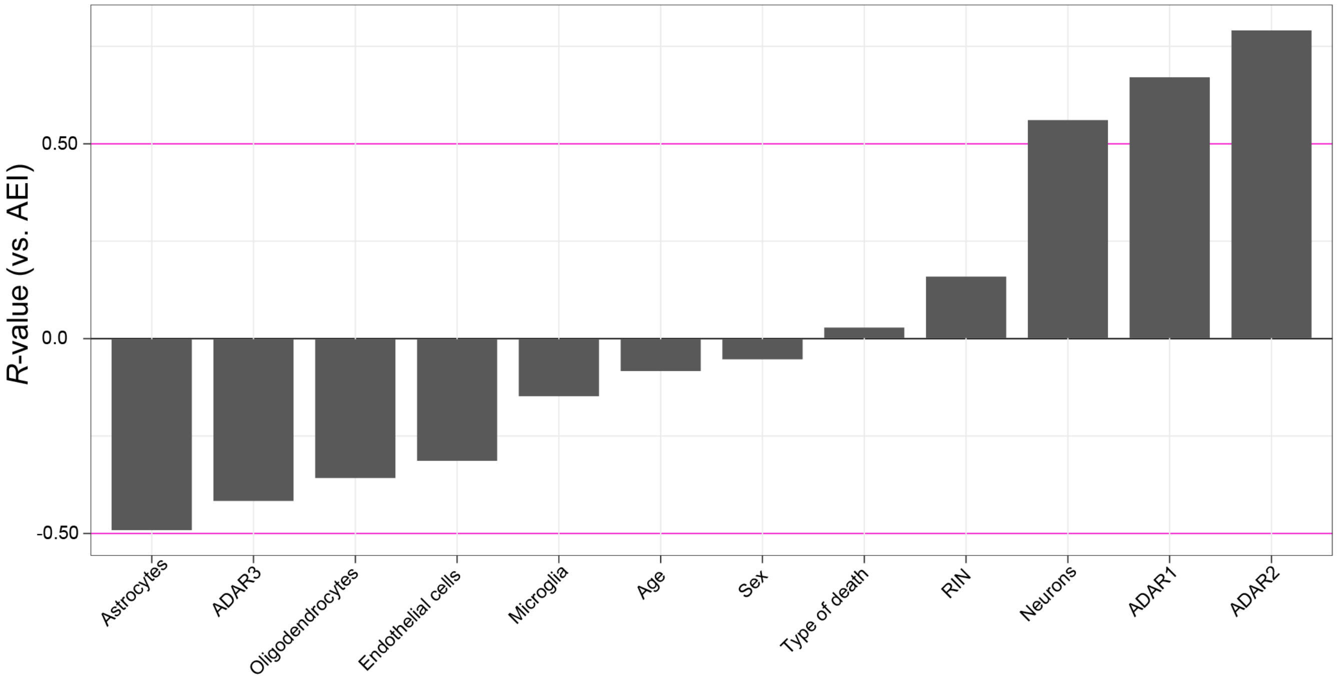

Supplementary Figure 13. Correlation of AEI with known factors in GTEx data. The AEI was correlated with 12 biological factors (x-axis), including estimated cell type proportions, using a Pearson's correlation coefficient (*R*, y-axis). Correlations were performed across 13 distinct GTEx brain regions. *ADAR2*, *ADAR1* and neuronal proportions display the strongest positive associations with AEI whereas non-neuronal cell types and *ADAR3* display the strongest negative associations with the AEI. The total number of independent biological replicates for each GTEx brain regions are reported in Supplementary Table 9.

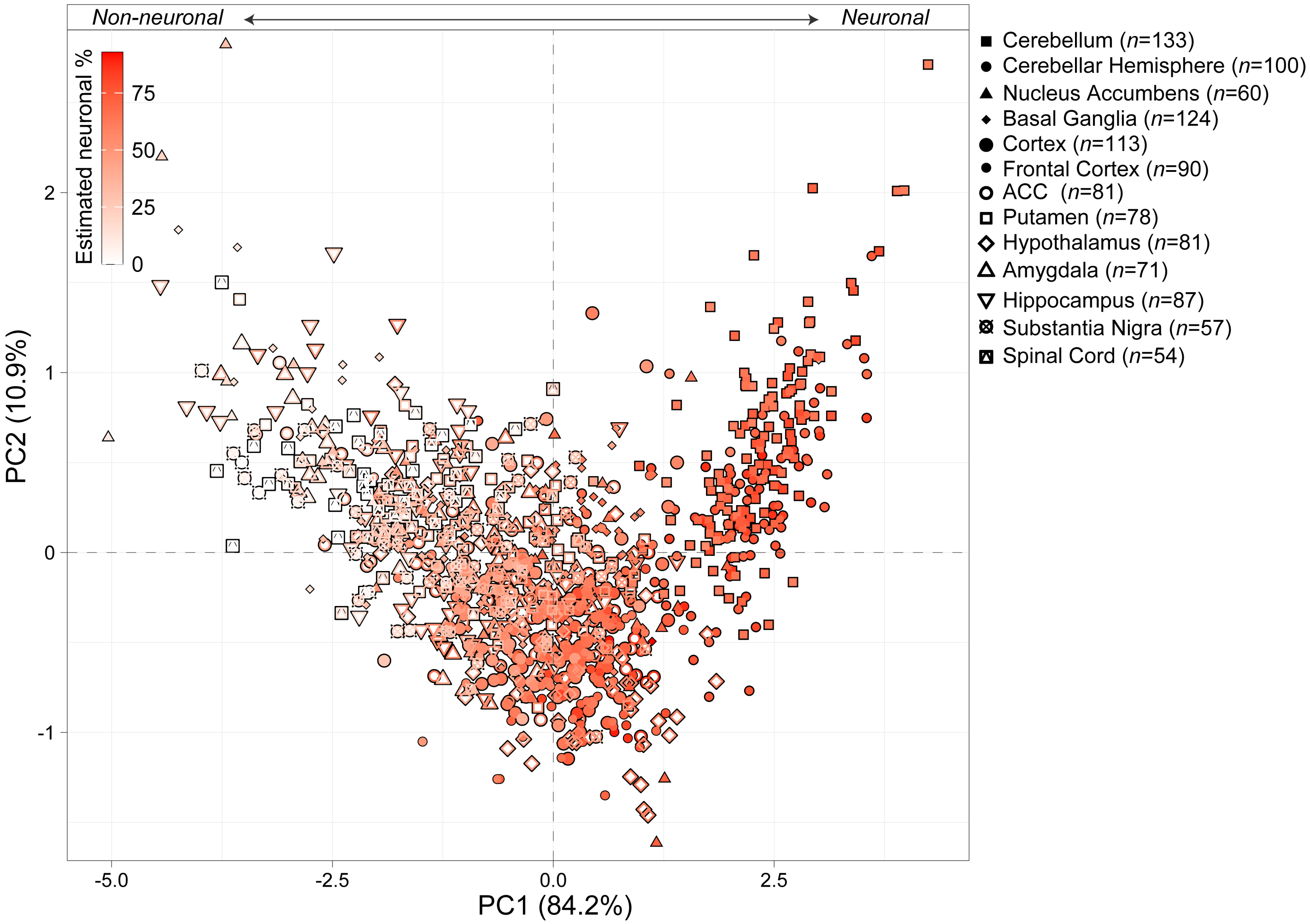

Supplementary Figure 14. Principal component analysis on features of RNA editing in the brain. We performed PCA on *ADAR1*, *ADAR2* and the AEI for each sample across thirteen GTEx brain regions. We then color coded each sample by the proportions of neurons per donor (%), from red- (high, more neuronal) to-white (low, less neuronal). This result indicates that PC1 (based on features of RNA editing in bulk brain tissues) is largely associated with samples and brain regions enriched with neurons. Sample sizes for each GTEx brain region are reported in the outer right panel.

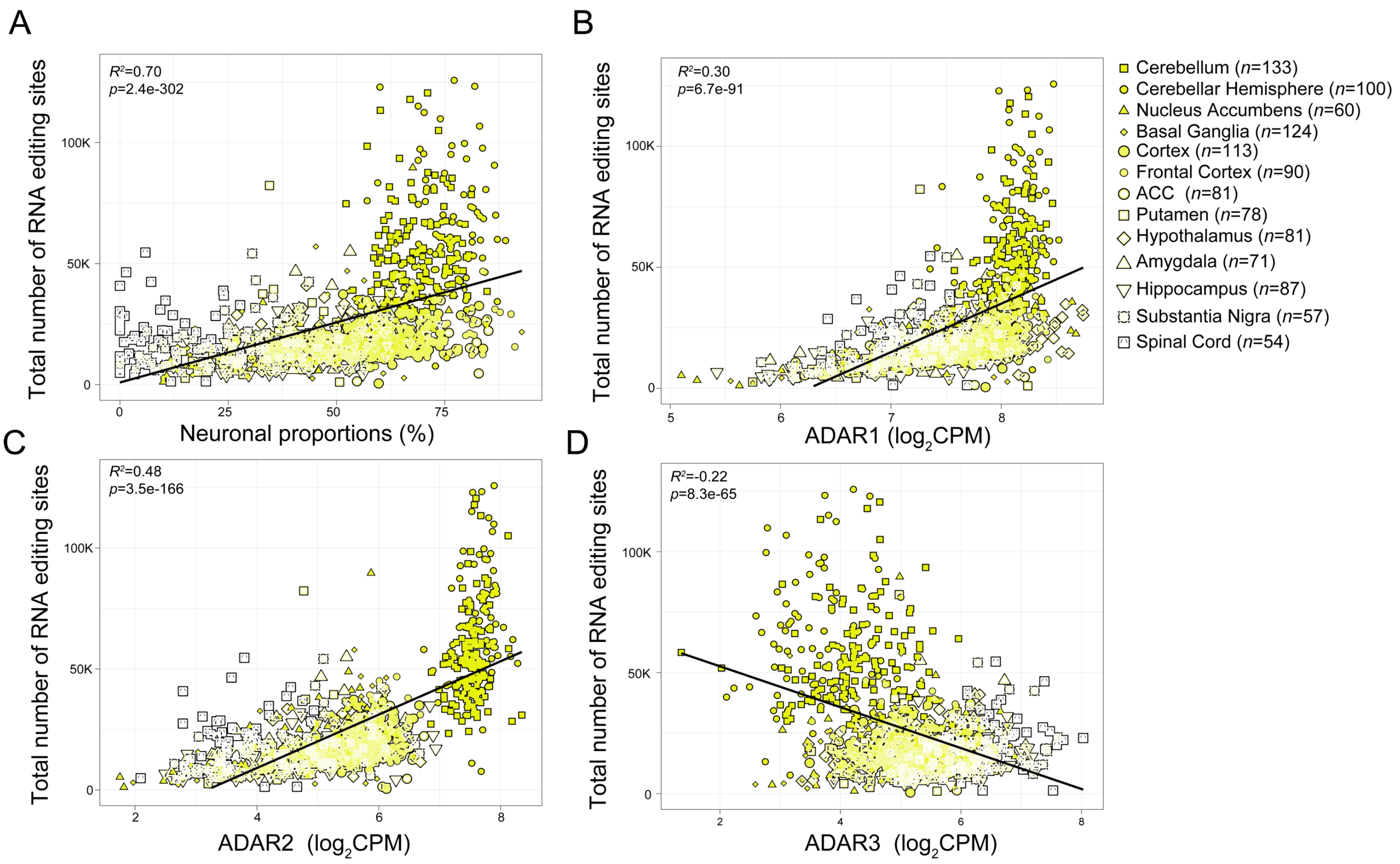

Supplementary Figure 15. Variance in RNA editing site detection per donor in bulk GTEx tissues. The amount of variance explained in the total number of RNA editing sites detected (y-axis) across all brain regions (denoted by point shape) according to differences in (**A**) neuronal proportions, (**B**) *ADAR1*, (**C**), *ADAR2*, (**D**) and *ADAR3* expression. Pearson's correlation coefficient was used to test each association and *R*-squared values and corresponding *p*-values are displayed. No adjustments were made for multiple comparisons. The total number of independent biological replicates for each GTEx brain region are reported in the outer right panel.

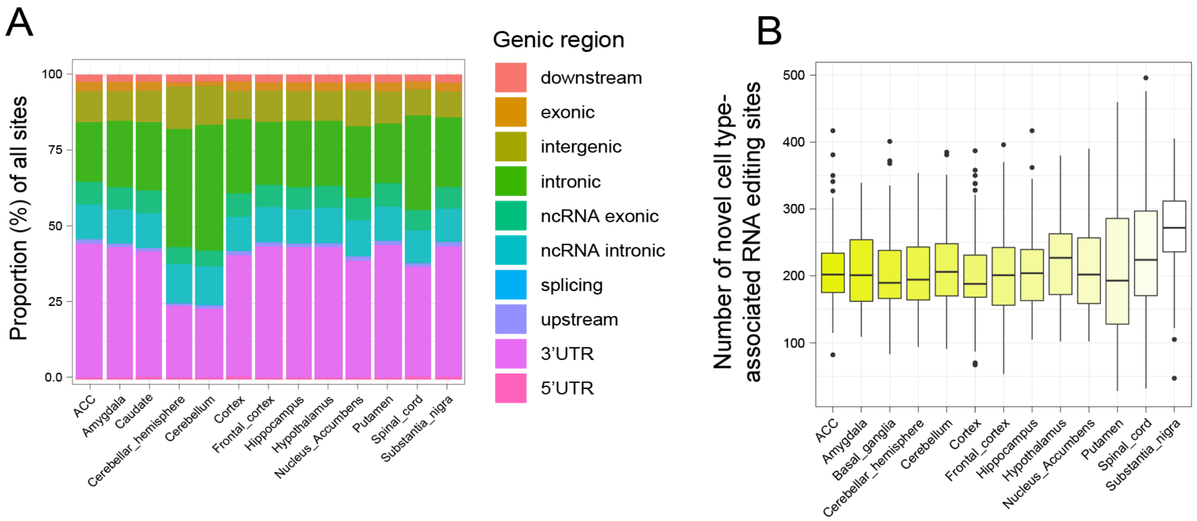

Supplementary Figure 16. Annotation of sites detected in bulk GTEx brain tissue. (**A**) The fraction of RNA editing sites per genic region according to brain regional differences. (**B**) The number of novel cell type-associated RNA editing sites detected in bulk brain RNA-sequencing data across all regions (~213 novel sites per region were detected). Box-and-whisker plots show the medians (horizontal lines), upper and lower quartiles (box edges), and 1.5 × the interquartile range (whiskers). Sample sizes for GTEx brain regions are reported in Supplementary Table 9.

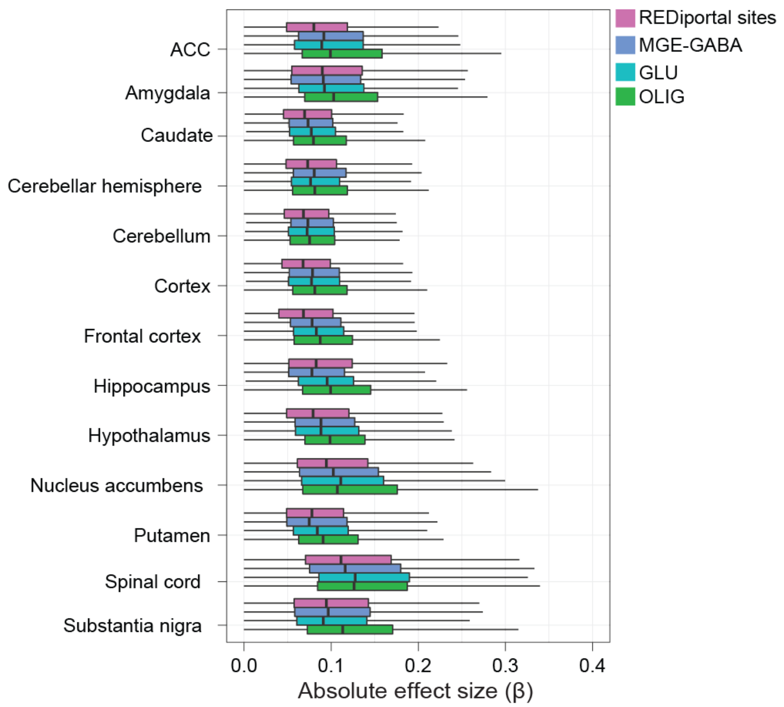

Supplementary Figure 17. Absolute effect sizes of edQTLs across GTEx brain regions. Independent boxplots for each brain region express that the absolute effect sizes (β) are consistently higher for edQTLs related to cell-associated editing sites and lower for all other edQTLs for editing sites without cellular annotation. Box-and-whisker plots show the medians (vertical lines), upper and lower quartiles (box edges), and 1.5 × the interquartile range (whiskers). Sample sizes for GTEx brain regions are reported in Supplementary Table 9.

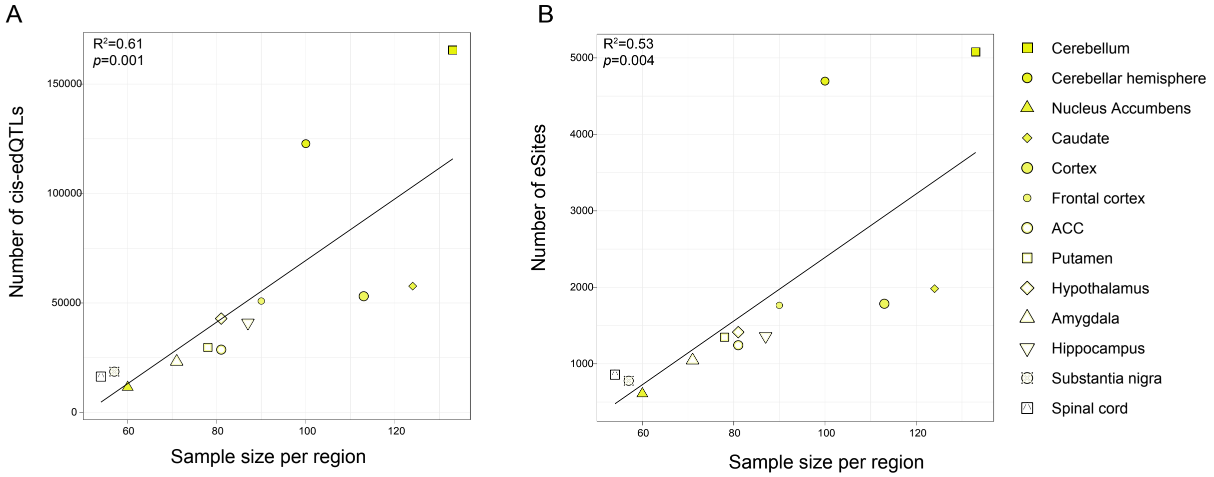

Supplementary Figure 18. edQTL discovery as a function of sample size. The total number of **(A)** cis-edQTLs (y-axis) **(B)** and eSites (y-axis) as a function of sample size (x-axis) for each brain region. Pearson's correlation coefficient was used to test each association and *R*-squared values and corresponding *p*-values are displayed. No adjustments were made for multiple comparisons. The total number of independent biological replicates for each GTEx brain regions are reported in Supplementary Table 9.

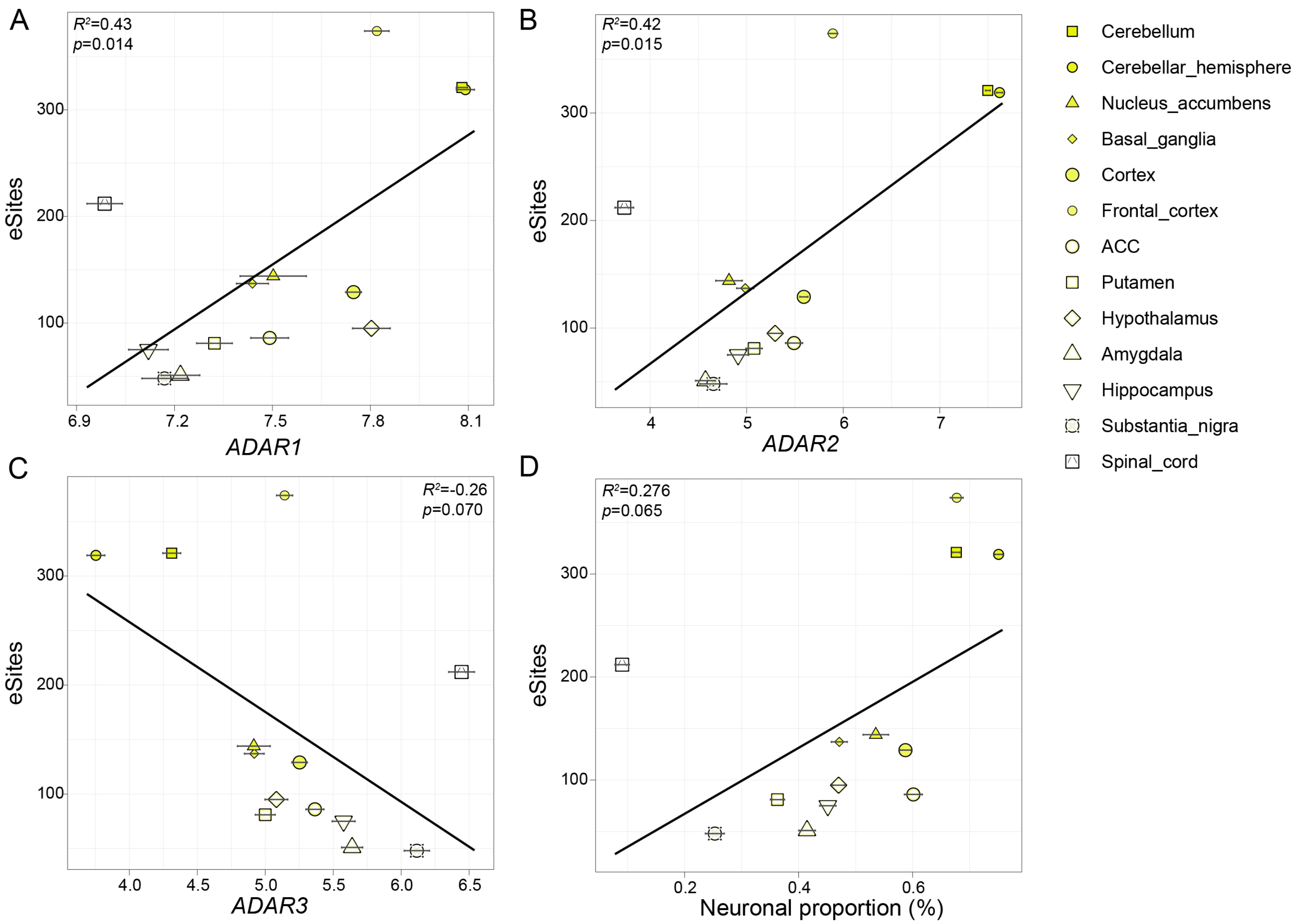

Supplementary Figure 19. eSite discovery in Park et al., 2021. eSite discovery per region pulled from Park et al., 2021 as a function of **(A)** *ADAR1* **(B)** *ADAR2*, (**C**) *ADAR3*, and (**D**) neuronal content. Data are presented as mean values +/- SEM all donors per region. Pearson's correlation coefficient was used to test each association and *R*-squared values and corresponding *p*-values are displayed. No adjustments were made for multiple comparisons. Sample sizes are reported as in Park et al., 2021.
