# Supplementary material for: Cellular and genetic drivers of RNA editing variation in the human brain
Source: Nat Commun. 2022 May 30;13:2997. doi: 10.1038/s41467-022-30531-0 (PMC9151768; doi:10.1038/s41467-022-30531-0)
Supplement: Supplementary file 3 — Description of Additional Supplementary Files [file 41467_2022_30531_MOESM3_ESM.pdf]

Description of Additional Supplementary Files

File Name: Supplementary Data 1
Description: *Alu* editing index and hyper-editing across purified cortical cell types.

File Name: Supplementary Data 2
Description: Annotation of cell-specific RNA editing sites.

File Name: Supplementary Data 3
Description: RNA editing sites as a function of gene length.

File Name: Supplementary Data 4
Description: RNA editing sites associated with ADAR and RBP expression.

File Name: Supplementary Data 5
Description: Differential RNA editing across cell types.

File Name: Supplementary Data 6
Description: Sample metadata and primer information for rhAmpSeq validation of recoding RNA-editing sites.

File Name: Supplementary Data 7
Description: RNA editing recoding events.

File Name: Supplementary Data 8
Description: snRNA-seq cellular pools.

File Name: Supplementary Data 9
Description: Features of global editing across bulk GTEx brain regions.

File Name: Supplementary Data 10
Description: Enrichment of cell-specific sites across GTEx brain regions.

File Name: Supplementary Data 11
Description: Max-edQTLs across bulk GTEx brain regions.
