# Supplementary material for: Cellular and genetic drivers of RNA editing variation in the human brain
Source: Nat Commun. 2022 May 30;13:2997. doi: 10.1038/s41467-022-30531-0 (PMC9151768; doi:10.1038/s41467-022-30531-0)
Supplement: Supplementary file 15 — Reporting Summary [file 41467_2022_30531_MOESM15_ESM.pdf]

nature portfolio

Corresponding author(s): Michael S. Breen, PhD

Last updated by author(s): Apr 12, 2022

Reporting Summary

Nature Portfolio wishes to improve the reproducibility of the work that we publish. This form provides structure for consistency and transparency in reporting. For further information on Nature Portfolio policies, see our Editorial Policies and the Editorial Policy Checklist.

Statistics

For all statistical analyses, confirm that the following items are present in the figure legend, table legend, main text, or Methods section.

n/a	Confirmed	
☐	☒	The exact sample size (*n*) for each experimental group/condition, given as a discrete number and unit of measurement
☐	☒	A statement on whether measurements were taken from distinct samples or whether the same sample was measured repeatedly
☐	☒	The statistical test(s) used AND whether they are one- or two-sided<br>*Only common tests should be described solely by name; describe more complex techniques in the Methods section.*
☐	☒	A description of all covariates tested
☐	☒	A description of any assumptions or corrections, such as tests of normality and adjustment for multiple comparisons
☐	☒	A full description of the statistical parameters including central tendency (e.g. means) or other basic estimates (e.g. regression coefficient) AND variation (e.g. standard deviation) or associated estimates of uncertainty (e.g. confidence intervals)
☐	☒	For null hypothesis testing, the test statistic (e.g. *F*, *t*, *r*) with confidence intervals, effect sizes, degrees of freedom and *P* value noted<br>*Give P values as exact values whenever suitable.*
☒	☐	For Bayesian analysis, information on the choice of priors and Markov chain Monte Carlo settings
☒	☐	For hierarchical and complex designs, identification of the appropriate level for tests and full reporting of outcomes
☐	☒	Estimates of effect sizes (e.g. Cohen's *d*, Pearson's *r*), indicating how they were calculated

Our web collection on statistics for biologists contains articles on many of the points above.

Software and code

Policy information about availability of computer code

Data collection	No software was used to collect data
Data analysis	All code is available on GitHub: https://github.com/BreenMS/RNA-editing-in-CNS-cell-types. The following methods and versions were used in this work: STAR v2.7.3 for mapping RNA-seq files, featureCounts (v.2.0.3) was used to count RNA-seq reads from mapped bam files, RseqQC (v.2.6.3) (http://rseqc.sourceforge.net/) was used to generate read coverage plots, the bMIND R package (v1.0) was used to perform cellular deconvolution of bulk brain tissue RNA-seq, Reditools v2.0 (parameters: -S -s 2 -ss 5 -mrl 50 -q 10 -bq 20 -C -T 2 –os 5) for de novo calling of RNA editing sites from mapped bam files, REDIportal database (2021) to curate known editing sites, ANNOVAR (v20211019) was used to annotated sites to genic regions, RepeatMasker (v4.1.1) was used to identify repeat elements, PHAST package (archived 2020-03-03) to quantify conservation metrics, samtools (v1.12) mpileup to query known sites from mapped bam files, AEI method (v1.0) to compute the Alu Editing Index from a mapped bam file, RNA hyper-editing was quantified from unmapped bam files using a previously described techniques (Porath et al., 2017), EDLogo (v1.0) to quantify local sequence motifs, regioneR (v1.14.0) to compute overlaps between two lists of coordinates, limma (v3.38.3) was used to compute differential editing and differential gene expression, ToppFunn to annotate genes (https://toppgene.cchmc.org/enrichment.jsp), SynGO to test for synaptic gene enrichment (https://syngoportal.org/), GeneOverlap function in R v1.8.0 to compute pairwise overlaps, SpliceAI (v1.3.1) to predict the probability of a site to be splice altering, CellRanger (v1.1.0) was used to process snRNA-seq data, , matrixEQTL (v2.3) was used to compute edQTLs. Additional scripts used in this study are available on GitHub both here (https://github.com/BreenMS/RNA-editing-in-CNS-cell-types) and here (https://github.com/ryncuddleston/RNA-hyper-editing).

For manuscripts utilizing custom algorithms or software that are central to the research but not yet described in published literature, software must be made available to editors and reviewers. We strongly encourage code deposition in a community repository (e.g. GitHub). See the Nature Portfolio guidelines for submitting code & software for further information.

Data

Policy information about availability of data

All manuscripts must include a data availability statement. This statement should provide the following information, where applicable:

- Accession codes, unique identifiers, or web links for publicly available datasets
- A description of any restrictions on data availability
- For clinical datasets or third party data, please ensure that the statement adheres to our policy

Raw RNA-sequencing FASTQ files across MGE-GABA, GLU, and OLIG cell populations in this study have been deposited in the Synapse database under accession code syn12034263 [https://www.synapse.org/#!Synapse:syn12034263]. Cell-type specific RNA editing sites generated in this study are provided in the Supplementary Data 2. Raw snRNA-seq files in this study have been deposited in the Synapse database under accession code syn15672826 [https://www.synapse.org/#!Synapse:syn15672826]. Approved access can be obtained for GTEx data through dbGaP (phs000424.v8). Further, to promote the exchange of this information, we developed an interactive Rshiny app with an easily searchable interface to act as a companion site for this paper: https://breenms.shinyapps.io/CNS_RNA_Editing/. Additional scripts used in this study are available on GitHub both here (https://github.com/BreenMS/RNA-editing-in-CNS-cell-types) and here (https://github.com/ryncuddleston/RNA-hyper-editing).

Field-specific reporting

Please select the one below that is the best fit for your research. If you are not sure, read the appropriate sections before making your selection.

☒ Life sciences ☐ Behavioural & social sciences ☐ Ecological, evolutionary & environmental sciences

For a reference copy of the document with all sections, see nature.com/documents/nr-reporting-summary-flat.pdf

Life sciences study design

All studies must disclose on these points even when the disclosure is negative.

Sample size	We used all FANS-derived RNA-seq and GTEx brain RNA-seq data available. No sample size calculation was needed.
Data exclusions	The focus of this study is the human brain. Therefore, we leveraged GTEx bulk RNA-seq data, and focused primarily on postmortem brain tissue and other non-brain tissues were pre-established as exclusionary for in the current study.
Replication	We validated FANS-derived RNA editing sites in independent snRNA-seq data from the DLPFC, in three independent biological replicates. Given the difference between technologies, we were able to validate ~8-27% of all cell-specific editing sites originally identified in FANS data.
Randomization	No randomization was performed for the current study as there are not multiple experimental groups in this study. Covariates were modeled using a linear mixed effect model to identify technical/biological factors that explain > 2% of editing variation in a given data frame. If meeting this criteria, these were added into our regression models as covariates and adjusted for in downstream analyses.
Blinding	Data was generated prior to conception of the current study, so blinding was not relevant.

Reporting for specific materials, systems and methods

We require information from authors about some types of materials, experimental systems and methods used in many studies. Here, indicate whether each material, system or method listed is relevant to your study. If you are not sure if a list item applies to your research, read the appropriate section before selecting a response.

Materials & experimental systems

n/a	Involved in the study	
☒	☐	Antibodies
☒	☐	Eukaryotic cell lines
☒	☐	Palaeontology and archaeology
☒	☐	Animals and other organisms
☒	☐	Human research participants
☒	☐	Clinical data
☒	☐	Dual use research of concern

Methods

n/a	Involved in the study	
☒	☐	ChIP-seq
☒	☐	Flow cytometry
☒	☐	MRI-based neuroimaging
